# Sensitivity to endothelin-1 is decreased in isolated livers of endothelial constitutive nitric oxide synthase knockout mice

**DOI:** 10.1186/1476-5926-5-9

**Published:** 2006-12-05

**Authors:** Andrea De Gottardi, Erwin Biecker, Abraham Koshy, Dieter Bohler, Sidney Shaw, Hans Sägesser, Jürg Reichen

**Affiliations:** 1 Department of Clinical Pharmacology, University of Berne, Murtenstrasse 35, 3010 Berne, Switzerland; 2 Division of Gastroenterology and Hepatology, University Hospital of Geneva, Rue Micheli-du-Crest 24, 1211 Geneva 14, Switzerland

## Abstract

**Background:**

Hepatic sinusoidal resistance is regulated by vasoactive factors including endothelin-1 (ET-1) and nitric oxide (NO). In the absence of NO, vasoconstrictor response to endothelin is expected to predominate. Therefore, we hypothesized sensitivity to endothelin to be increased in mice lacking the endothelial cell NO synthase gene. Response of vascular resistance to endothelin was assessed in the *in situ *perfused liver of endothelial constitutive nitric oxide synthase (ecNOS) knockout and wild type mice. Livers were also harvested for RNA and protein isolation for quantitative PCR and Western blotting, respectively. The expression of endothelin receptors, isoenzymes of NO synthase, heme-oxygenase and adrenomedullin was quantified.

**Results:**

Endothelin increased hepatic vascular resistance in a dose-dependent manner in both strains; however, this increase was significantly less in ecNOS knockout mice at physiologic concentrations. Expression of heme-oxygenases and adrenomedullin was similar in both groups, whereas inducible nitric oxide synthase (iNOS) protein was not detectable in either strain. mRNA levels of pre-pro-endothelin-1 and ET_B _receptor were comparable in both strains, while mRNA for ET_A _receptor was decreased in ecNOS knockouts.

**Conclusion:**

Livers of ecNOS knockout mice have a decreased sensitivity to endothelin at physiologic concentrations; this is associated with a decreased expression of ET_A _receptors, but not with other factors, such as iNOS, ET_B _receptors, adrenomedullin or heme-oxygenase. Further studies targeting adaptive changes in ET_A _receptor distribution and/or intracellular signaling downstream of the receptor are indicated.

## Background

Sinusoidal perfusion is highly variable and regulated by different humoral substances including nitric oxide and endothelin [1-3]. Endothelin-1 (ET-1), one of the most potent endogenous vasoconstrictors [4], has extra- and intra-sinusoidal actions, the latter being more important at low endothelin concentrations [1,5] This effect has been associated to hepatic stellate cell contraction [6]. The resulting increase in shear stress activates endothelial nitric oxide (NO) production *via *ET_B _receptors [7].

Intrahepatic vascular resistance is also regulated by vasoactive substances that may act locally or systemically. An excess of vasoconstrictors increases the vascular tone and may lead to an exaggerated response of the hepatic vascular bed. These factors include noradrenaline, angiotensin II and leukotrienes [8], but ET-1 seems to be the most potent one. In the rat liver, the ET_A _receptor subtype causes vasoconstriction, while the ET_B _receptor subtype is associated with a dual vascular response. ET_B _on hepatic stellate cells mediates their constriction, but this is normally countered by the vasodilatory effect of NO, released under the regulation of ET_B _on endothelial sinusoidal cells [1].

The vascular balance is maintained by the availability of vasodilators. NO is the best known, but other molecules such as carbon monoxide [9] and adrenomedullin contribute to intrahepatic vasodilation [10].

The release of NO has been demonstrated to be a crucial regulatory mechanism counteracting the action of ET-1 in the kidney of ET-1 transgenic mice, highlighting the *in vivo *interaction between NO and ET-1 [11]. Additionally, an increased NO bioavailability has been shown to improve endothelium-dependent relaxation of aortic rings from mice overexpressing ET-1, suggesting that, in the presence of an activated ET system, NO production may essentially contribute to maintain a normal vascular pressure [12].

Mice in which the key enzyme catalyzing the release of NO, endothelial constitutive NO synthase, has been knocked out have arterial hypertension but are otherwise phenotypically normal [13]. We argued that the hepatic vasculature of such mice should be more sensitive to exogenous endothelin-1 since the compensatory vasodilatation could not occur. Therefore, vascular resistance in response to ET-1 was studied in the perfused mouse liver of ecNOS knockout mice and their wild type counterparts.

Surprisingly, sensitivity to ET-1 was decreased in ecNOS knockout mice, suggesting the presence of compensatory mechanisms counteracting the absence of NO. The decreased hepatic expression of ET_A _receptors in endothelial constitutive nitric oxide synthase (ecNOS) knockout mice may contribute to the observed decreased vascular sensitivity.

## Results

### General observations

**Figure 1:**
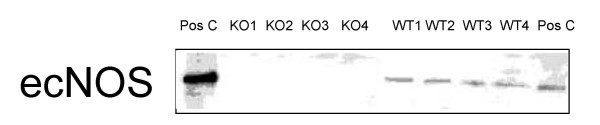
Western blot of ecNOS in wild-type and ecNOS knockout mice. The enzyme was not detectable in the knockouts. Data are from single mice and not from pools.

**Figure 2:**
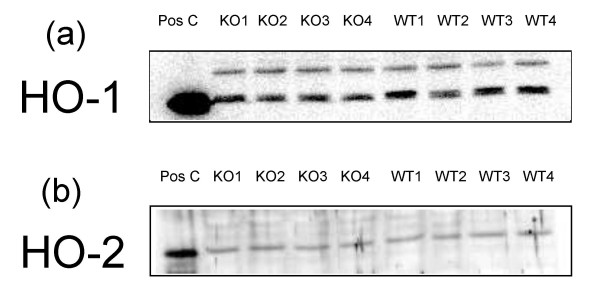
Western blot of heme oxygenase 1 (2a) and 2 (2b) in wild-type and ecNOS knockout mice. There was no difference in expression between the two strains. Data are from single mice and not from pools.

All animals were tested for the presence of ecNOS. On Western blots ecNOS was detectable in wild type, but not in knockout mice (Fig. 1). The same held true for ecNOS mRNA as demonstrated by real time quantitative PCR (data not shown). We tested whether iNOS or the two isoenzymes of heme oxygenase would compensate for the lack of ecNOS. Inducible NOS was not detectable by Western blotting in either strain [14] while the expression of heme oxygenase 1 and 2 did not differ between the two strains (Figs. 2a, 2b). Heme oxygenase 1 in wild type and ecNOS knockout mice were 42 ± 11 and 42 ± 9 Linear Arbitrary Units (LAU), respectively. Heme oxygenase 2 in wild type and ecNOS knockout mice were 193 ± 52 and 171 ± 45 LAU, respectively. Furthermore, adrenomedullin mRNA was probed by real-time PCR to investigate whether it would be upregulated in the absence of NO; there was no difference between the two groups, ΔC_T _averaging 11.0 ± 0.5 and 11.1 ± 0.4 in wild-type and knockout mice, respectively.

### Effect of ET-1 on portal resistance

**Figure 3:**
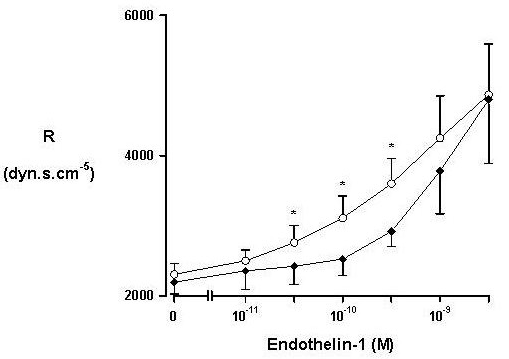
Resistance in the *in situ *perfused mouse liver of wild type (○) and ecNOS knockout (◆) mice in response to endothelin 1. Mean and one standard deviation are shown. * Denotes a statistically significant difference, as assessed by ANOVA.

ET-1 induced a dose-dependent increase in portal perfusion pressure and a decrease in portal flow in both mouse strains; this resulted in a dose-dependent increase in hepatic vascular resistance (Fig. 3). There was an increase in hepatic resistance in both strains; however, this increase was significantly less marked at concentrations 3 × 10^-10 ^to 3 × 10^-9 ^M in ecNOS knockout mice. At higher, non-physiologic concentrations, there was no difference between ecNOS knockout and wild type mice. Viability of the perfused organ as assessed by K^+ ^and alanine aminotransferase (ALT) release into the perfusate was not affected in either strain (data not shown).

### Expression of pre-pro-endothelin-1 and its receptors

ET-1 radioimmunoassay showed no significant difference between the two groups, being 7 ± 6 and 12 ± 20 pg/100 mg liver tissue in wild-type and knockout mice, respectively. The mRNA steady state levels, quantified by real-time PCR, of pre-pro-endothelin-1 and ET_B _receptor were comparable in WT and ecNOS knockout mice (Table 1). In contrast, mRNA of ET_A _receptors was reduced by 65% in ecNOS knockout mice compared to WT mice (Table 1).

**Table 1:** Expression of ET-1; ET_A _and ET_B _receptors in the liver of wild-type and ecNOS knock-out mice.

	Wild-type	ecNOS knock-out
mRNA for ET-1	14.8 ± 0.5	15.0 ± 0.2
mRNA for ET_A_†	9.9 ± 0.3	11.4 ± 0.3*
mRNA for ET_B_	11.5 ± 0.7	11.9 ± 0.3

All results are expressed as mean ± standard deviation (n = 6 animals per group). The two animal groups were compared by Student's t-test. † the 1.5 cycle increase in ET_A _mRNA in ecNOS knock-out mice indicates a 65% decrease in mRNA compared to wild-type mice. * *p *= 0.0005.

## Discussion

Contrary to our expectations, the present investigation demonstrates a decreased sensitivity of the liver from ecNOS knockout mice to exogenous ET-1. This appears to be achieved independent of changes in other major vasodilatory systems including inducible nitric oxide synthase (iNOS), heme oxygenase and adrenomedullin.

Different factors that could compensate for the lack of ecNOS were evaluated. The prime candidate being obviously the inducible isoform [15], it was not detectable by Western blotting excluding its participation in the reduced response to endothelin in ecNOS knock-out mice. Direct assessment of NO formation by the citrulline assay [16] is not reliable in liver tissue owing to competing enzymatic reactions. Hence, other potential candidates were examined.

Carbon monoxide (CO) has recently been described as another factor modulating sinusoidal tone [9]. NO and CO may complement each other as signaling molecules in some physiological situations [17-19]. Heme oxygenase catalyses the production of CO from heme [17]. However, heme oxygenase isoforms 1 and 2 were similarly expressed in the two strains on the protein and mRNA level, making unlikely a contribution from this system. The same held true for adrenomedullin, a potent vasodilator peptide [20] which acts in part through cAMP and in part through ecNOS [21-26]. However, decreased NO may theoretically reduce adrenomedullin receptor availability even if it increases adrenomedullin mRNA, since in a rat mesangial cell culture system, NO donors increased binding of adrenomedullin to its receptor but reduced adrenomedullin mRNA levels [27].

Finally, we looked at the endothelin system itself. Although endothelin was originally described as a potent vasoconstrictor [4], depending on the particular receptor involved it can also have vasodilatory properties. It would have been conceivable that endothelin would be altered in the absence of ecNOS-derived NO; this was clearly not the case since at the protein and the mRNA level there was no difference between the two strains.

Vasoconstriction is mediated mainly by ET_A _receptors [28], but also by ET_B _receptors present on smooth muscle cells [29]. In contrast, vasodilatation is evoked via ET_B_receptors through release of endothelium derived vasodilators such as NO and prostacyclin [30,31] ET_B_receptor mRNA was unchanged as shown by quantitative PCR. This suggests that ET_B _receptors did not contribute to adaptive changes in ecNOS knockout mice. Furthermore, ET_B _receptors are not expected to contribute to vasodilatation in ecNOS knockout mice as the vasodilatory action of ET_B _receptors is mediated predominantly through ecNOS.

In the ecNOS knockout mice, the expression of ET_A _receptors was down-regulated. This observation may explain the attenuated response to ET-1 in ecNOS knockout mice and makes biological sense as an adaptive mechanism. In fact, under basal conditions the portal pressure in wild type and ecNOS knockout mice is similar, suggesting that a decrease in vasoconstrictor mechanisms, in our model the decrease of the expression of ET_A _receptors, compensates for a decrease in vasodilator factors, in our model the lack of ecNOS. However, this equilibrium is not maintained when a vasoactive stress, such as the perfusion of the liver with ET-1, is applied. The use of the perfused liver model allowed us to postulate that, in ecNOS knockout mice, the decreased sensitivity to ET-1 is associated to an insufficient expression of ET_A _receptors. Investigation of the mechanisms by which ET_A _receptors contribute to the unexpected decreased sensitivity of liver from ecNOS knockout mice to exogenous endothelin-1 was beyond the scope of this study. In a recent study, the expression of ET_A _receptors was decreased in endothelium-denuded aortae of ecNOS KO mice, suggesting an adaptive mechanism similar to the one we observed. Nevertheless, in these animals, an increased expression of cyclooxygenase-2 overcame the decrease of ET_A _and enabled and increased sensitivity to ET-1 [32].

## Conclusion

This study has demonstrated the capacity of adaptation of the endothelin system in a model of perfused isolated liver in ecNOS KO mice. The mechanisms involved in this adaptive response involve a decreased hepatic expression of the ET_A _receptor in the absence of NO, leading to an attenuation of the vasoconstriction induced by ET-1. These data suggest that ET_A _may be one major player in the regulation of hepatic vascular resistance. The expression of ET_A_, which is increased in cirrhotic livers [33], may hence represent a major pharmacological target to ameliorate portal pressure due to the imbalance between vasoconstrictive and vasodilative mechanisms. Experimental data on the use of ET_A _blockers in an animal model of portal hypertension corroborate this hypothesis [34].

## Methods

### Animals

ecNOS knockout mice [13] were obtained from Dr. P. L. Huang and bred locally. Wild type mice of the same genetic background (C57BL/6J) served as controls. The mice, all of male sex, were kept under a 12 h dark-light cycle with free access to mouse chow and drinking water. The protocol was approved by a state board on animal experimentation; all experiments were performed according to international guidelines concerning the conduct of animal experimentation.

### In situ liver perfusion

On the day of experimentation, mice were anesthetized with pentobarbital (70 mg/kg), intra-peritoneal. Five each of knockout and wild type animals were studied. Mouse liver perfusion was carried out *in situ *as described previously from these laboratories using a pressure head [35]. The perfusion medium consisted of Krebs-Ringer-bicarbonate buffer containing bovine serum albumin (2 % w/v) and dextrose (0.1 % w/v). After a warming up period of 20 minutes, baseline flow and pressure were recorded and resistance calculated. Then, ET-1 was infused at increasing concentrations (10^-11 ^to 3 × 10^-9 ^M), without recirculation; after 10 minutes, flow and pressure were recorded again. ALT and K^+ ^release into the media were recorded as a measure of viability of the perfused organ.

### Biochemical analyses

Six animals each of the wild-type and ecNOS knock-out group were anesthetized as described above. The livers were removed; half was homogenized for protein determination and the other half used for RNA preparation (*vide infra*). Homogenization was carried out in four volumes of 0.25 mol/L sucrose at 4°C. Protein concentration was determined according to Lowry [36].

### Western blot

Western blots were performed as previously reported [37]. In short, proteins from liver homogenate were separated by sodium dodecyl sulfate-polyacrylamide gel electrophoresis using a 5% polyacrylamide gel for NOS or 12% gel for HO and subsequently transferred to nitrocellulose membranes. The membranes were blocked overnight with BSA at 4°C and probed for 2 hours with the primary antibody. Antibodies against iNOS and ecNOS were obtained from Transduction Laboratories (Lexington KY, USA) and antibodies against HO1 and HO2 from StressGen Biotechnologies (Victoria, Canada). HO1 (Hsp32) recombinant protein, OSP-500 recombinant human HO2 protein (both from StressGen Biotechnologies) and mouse macrophage lysate iNOS (Transduction Laboratories) were used as positive controls. The membranes were washed twice with phosphate-buffered saline, incubated for 1 hour with peroxidase-conjugated secondary antibody (IgG anti-rabbit), and detected by enhanced chemiluminescence (ECL Western Blot kit from Amersham Life Science). Luminometric analysis of Western blots were performed on Luminescent image analyzer LAS-1000 (Fujifilm) and expressed as LAU.

### Radioimmunoassay of ET-1

Radioimmunoassay of ET-1 in liver tissue was as described from our laboratories [38]. Briefly, snap frozen tissue was homogenized in a chloroform-ethanol 2:1 solution with 0.1% trifluoroacetic acid and 1 mM N-ethylmaleamide. To each tube a volume of 40% of sterile water was added and centrifuged at 48°C, 3900 g for 15 min. The aqueous phase was collected, diluted 1:9 in acetic acid 4% and passed through activated Sep-Pak C18 500 mg cartridges (Waters Corporation, Milford, USA). The product of elution (2 ml 86% ethanol/4% acetic acid) was dried overnight in a Speed-Vac centrifuge system. Endothelin-1 was then analyzed by a double antibody radioimmunoassay technique. Endothelin-1 was obtained from Sigma (St. Louis, USA), ET-1 antibodies were from Peninsula (St. Helens, England), and [125I]-ET-1 was obtained from Amersham International (Buckinghamshire, UK).

### RNA extraction and quantitation

Total RNA was isolated using the guanidinium isocyanate method [39]. Five μg of total RNA were reverse transcribed in a final volume of 20 μL using the Moloney Murine Leukemia Virus Reverse Transcriptase (Gibco Life Technologies, USA). The Perkin-Elmer 7700 Sequence Detection System (Rotkreuz, Switzerland) for the quantitative polymerase chain reaction assay was used. This is based on the principle of detection of specific PCR products with fluorogenic probes [40,41]. as previously described from our laboratories [38]. Briefly, the probe contains a fluorescent reporter dye covalently linked to the 5' end, and a quencher dye linked close to the 3' end. The closeness of the quencher to the reporter emitter means that the reporter fluorescence is suppressed. During PCR cycling, the probe specifically hybridizes to the corresponding template and is then cleaved via the 5' to 3' exonuclease activity of Taq DNA polymerase. This cleavage results in an increase of fluorescence emission of the reporter dye proportional to the amount of specific PCR product. The sequences of the probes and primers were designed according to the manufacturer's guidelines and are reported in Table 2. The threshold cycle (C_T_) of the mRNA of interest (target) was expressed with reference to the C_T _of internal Glyseraldehyde 3-phosphate dehydrogenase (GAPDH) mRNA (ΔC_T _= target C_T _- GAPDH C_T_).

**Table 2:** Primers and probes used in real-time PCR.

**Gene**	**Forward primer**	**Probe**	**Reverse primer**
PreproET1	*tggtggaaggaaggaaactacg*	*aggttggaggccatcagcaacagc*	*ttgcaacacgaaaagatgcc*
ET_A_	*tgacctccccatcaacgtg*	*ttaagctcttggcaggacgctggc*	*tccaaaatcattgtggtcgaaa*
ET_B_	*cgtgttcgtgctaggcatca*	*cgggaactccacgctgctaagaatcat*	*ttgcgcatgcacttgttctt*
GAPDH	*actggcatggccttccg*	*ttcctacccccaatgtgtccgtcgt*	*caggcggcacgtcagatc*
ecNOS	*ctgcaaaccgtgcagagaatt*	*tggcaacagagggcggcatg*	*caccggcttcatccagct*
iNOS	*ggcagcctgtgagacctttg*	*tgtccgaagcaaacatcacattcagatcc*	*ttgcattggaagtgaagcgtt*
Adrenomedullin	*cagggttcccgcagca*	*atgccgcttcgggacctgca*	*tagatctggtgggccaatttct*

### Statistical analysis

All results are reported as mean ± 1 standard deviations. Means of the two groups were compared by Student's t-test. Normality was verified by studying normal quantile plots (QQ plots). Dose-response curves were analyzed by analysis of variance with regression. A *p *< 0.05 was considered statistically significant.

## Competing interests

The author(s) declare that they have no competing interests.

## Authors' contributions

ADG carried out parts of the animal experiments and bench work and prepared the manuscript. EB, AK, DB and HS contributed to the animal experiments and critically revised the manuscript. SS provided assistance for the endothelin expression studies and for the preparation of the manuscript. JR contributed to the design of the study, obtained funding for the study and critically revised the manuscript. All authors read and approved the final manuscript.
